# Antibacterial, Antibiofilm and Anti-Virulence Activity of Biactive Fractions from Mucus Secretion of Giant African Snail *Achatina fulica* against *Staphylococcus aureus* Strains

**DOI:** 10.3390/antibiotics10121548

**Published:** 2021-12-17

**Authors:** Libardo Suárez, Andrés Pereira, William Hidalgo, Nelson Uribe

**Affiliations:** 1 Grupo de Investigación en Bioquímica y Microbiología (GIBIM), Escuela de Química, Universidad Industrial de Santander, Edificio Camilo Torres 202, Bucaramanga 680002, Colombia; libardo2178128@correo.uis.edu.co; 2 Grupo de Inmunología y Epidemiología Molecular (GIEM), Escuela de Microbiología, Facultad de Salud, Universidad Industrial de Santander, Bucaramanga 680002, Colombia; andres.pereira@correo.uis.edu.co (A.P.); nelurdel@uis.edu.co (N.U.)

**Keywords:** *Achatina fulica*, biofilm, antibacterial activity, *Staphylococcus aureus*

## Abstract

*Staphylococcus aureus* is an important etiological agent that causes skin infections, and has the propensity to form biofilms, leading to significant mortality and morbidity in patients with wounds. Mucus secretion from the Giant African snail *Achatina fulica* is a potential source of biologically active substances that might be an important source for new drugs to treat resistant and biofilm-forming bacteria such as *S. aureus*. This study evaluated the effect of semi-purified fractions from the mucus secretion of *A. fulica* on the growth, biofilm formation and virulence factors of *S. aureus*. Two fractions: FMA30 (Mw >30 kDa) and FME30 (Mw 30−10 kDa) exhibited antimicrobial activity against *S. aureus* with a MIC_50_ of 25 and 125 µg/mL, respectively. An inhibition of biofilm formation higher than 80% was observed at 9 µg/mL with FMA30 and 120 µg/mL with FME30. Furthermore, inhibition of hemolytic and protease activity was determined using a concentration of MIC_20_, and FME30 showed a strong inhibitory effect in the formation of clots. We report for the first time the effect of semi-purified fractions of mucus secretion of *A. fulica* on biofilm formation and activity of virulence factors such as α-hemolysin, coagulase and proteases produced by *S. aureus* strains.

## 1. Introduction

*Staphylococcus aureus* is an opportunistic pathogen and a leading cause of skin infections, as well as an important cause of pneumonia and bacteremia in both healthcare and community settings [1]. In addition, *S. aureus* has the propensity to form biofilms and attachment to medical implants and host tissue, playing an important role in the persistence of chronic infections [2].

The first methicillin-resistant strain of *S. aureus* was reported in 1960 and developed over time resistance to different antibiotics such as linezolid, daptomycin and glycopeptides [3]. For this reason, in 2017 the World Health Organization (WHO) included this microorganism in the “Global priority list of antibiotic-resistant bacteria to guide research, discovery, and development of new antibiotics” [4].

Research into new natural compounds with antimicrobial activity or that inhibit biofilm formation is a priority in the fight against antimicrobial resistance [4,5].

Phylum Mollusca, among other invertebrates, are a large reservoir for pharmacologically active compounds present in hemolymph and mucus secretions [6,7,8]. Mucus secretion from the Giant African snail *Achatina fulica* is a potential source of biologically active substances that might lead to the discovery of new drugs to treat resistant bacteria such as MRSA [9]. Phylum Mollusca, among other invertebrates, are a large reservoir for pharmacologically active compounds present in hemolymph and mucus secretions [6,7]. Mucus secretion from Giant African snail *Achatina fulica* is a potential source of biologically active substances that might lead to the discovery of new drugs to treat resistant bacteria such as MRSA [9].

*A. fulica* is a gastropod native to eastern Africa, reported in more than 60 countries on five continents, and included in the *100 of the World’s Worst Invasive Alien Species*, due to the impact on agriculture, ecosystems, human health and economy [10]. Despite of some studies on the mucus of this snail that have shown different biological activities such as inhibition of microbial growth, inhibition of inflammatory processes, and healing properties, there is still little information available about antimicrobial compounds and biofilm formation inhibitors from *A. fulica* and terrestrial gastropods in general [11,12,13].

The antibacterial effect of the mucus of *A. fulica* seems to be related to the presence of proteins and antimicrobial peptides (AMPs). Nevertheless, aside from the L-amino acid oxidase *Achacin* (59 kDa) or the cysteine-rich AMP *mytimacin-AF* (9.7 kDa), few antimicrobial compounds have been fully identified and characterized [7,12]. 

Hemocyanin is one of the most important proteins present in the hemolymph of crustaceans, arachnids and mollusks that is responsible for the transport of oxygen. Its function is equivalent to that of hemoglobin in humans. So far, hemocyanin has only been found in two phyla of the animal kingdom: in mollusks and in arthropods. In mollusks it has been found in all cephalopods, and in arthropods in all decapods [14,15]. However, in these two phyla the distribution of hemocyanin is erratic, for example, in the snail of the genus *Helix* it is found in large quantities, but in the freshwater species *Planorbis*, no hemocyanin has been found. Likewise, hemocyanin has been found in certain types of scorpions and spiders [15,16].

The interest of hemocyanin as a bioactive molecule against pathogenic microorganisms is relatively recent [17,18]. In the same way, the hemocyanin studied has been mainly from some species of shrimp, crabs and arachnids. For example, rondonin (1.2 kDa) is a peptide with antifungal activity against *Candida albicans* that inhibits its growth at a concentration of 67 mM. The rondonin sequence (IIIQYEGHKH) shows identity with the C-terminal fragment of the “d” subunit of hemocyanin from the *Eurypelma californicum* tarantula [16].

On the other hand, although in 2018 Ishwaryam et al. reported the antibiofilm activity of 78 kDa subunit of crustacean hemocyanin [19], the information on the antibiofilm and anti-virulence activity of terrestrial mollusks hemocyanin is still very scarce. Therefore, we consider that the study of not only purified subunits, but fractions with peptides derived from hemocyanin from other species of mollusks has interest and brings valuable information for novel studies in this area.

Here, we report the antimicrobial activity of fractions of *A. fulica* mucus against *S. aureus* strains and, for the first time, described is the effect of semi-purified fractions of mucus secretion on biofilm formation and anti-virulence activity on factors such as α-hemolysin, coagulase, and proteases produced by *S. aureus*.

## 2. Results

### 2.1. Purification and Characterization of Fractions from Mucus of Achatina fulica

**Figure 1:**
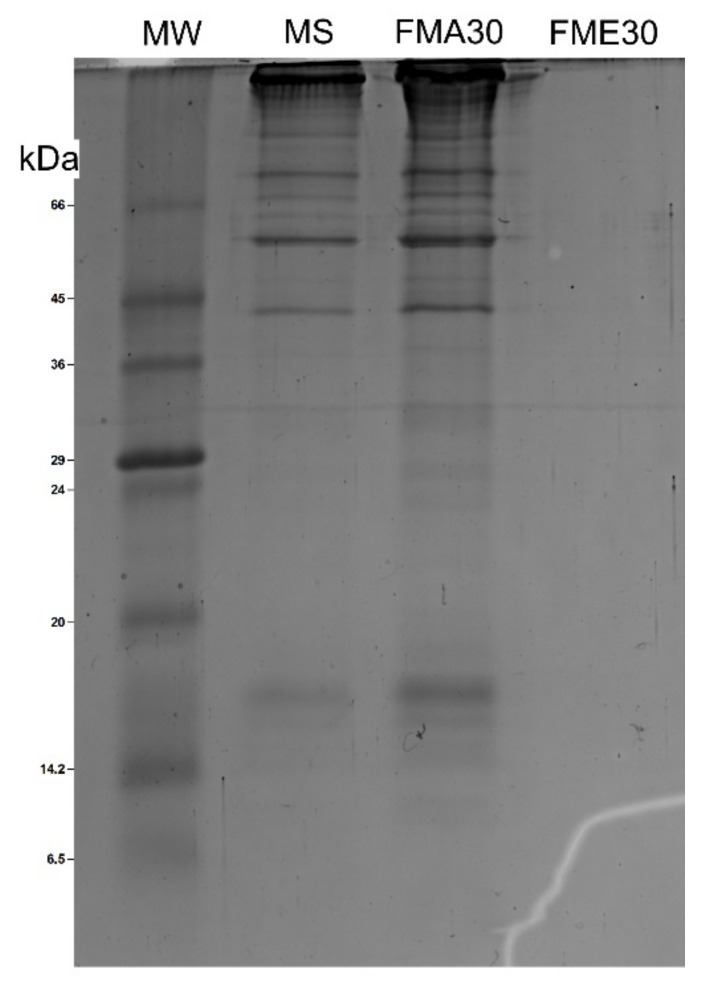
SDS-PAGE of separated fractions of mucus secretion of *A. fulica.* MW (molecular weight marker), MS (mucus secretion).

The purified crude mucus extract separated in three semi-purified fractions named FMA30 (Mw > 30 kDa), FME30 (Mw 30−10 kDa) and FME10 (Mw < 10 kDa) were obtained using ultra-centrifugal filters with pore size of 30 kDa and 10 kDa, although in the fraction less than 10 kDa, no bands on the SDS-PAGE gel were observed. For the fraction greater than 30 kDa, the same bands of approximately 20 kDa, 30 kDa, 40 kDa, 60 kDa and 80 kDa were constantly obtained (Figure 1).

Mass analysis revealed that the bands of approximately 20 kDa, 40 kDa, 60 kDa and 80 kDa were composed of peptides that are part of the hemocyanin protein. A total of 20 (Table 1) peptides were identified in FMA30 that support the presence of hemocyanin in the mucus secretion of *A. fulica*. Since the *A. fulica* hemocyanin sequence is not available in UniProt/SwissProt database for mollusks, the alignment of the peptides using the BLASTp tool confirmed their identity with hemocyanin from land snails *C. asperum*, *H. pomatia*, *H locrum*, the freshwater snail *L. stagnalis* and the marine gastropod *Aplysia californica*.

The mass analysis of FME30 showed two peptides that did not match entries in the UniProt/SwissProt database for mollusks and AMPs.

### 2.2. In Vitro Determination of MIC

The antimicrobial activity of fractions against *Staphylococcus* strains were determined by measuring MIC_50_ (Table 2). The fraction FMA30 exhibited the best antimicrobial activity against *S. aureus* CMPUJ 015 and *S. aureus* ATCC 29213 with a MIC_50_ of 25 and 125 µg/mL, respectively. Additionally, the sub-inhibitory MICs (Table 2) were determined, as they were necessary for further bioassays. Additionally, FME10 was evaluated (a fraction obtained with a filter of 10 kDa) but it did not exhibit biological activity (Figure S1).

### 2.3. In Vitro Inhibition of Biofilm Formation

**Figure 2:**
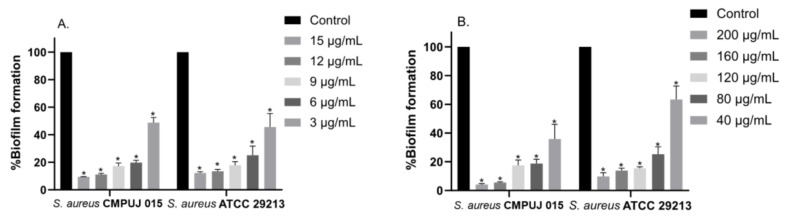
Antibiofilm effect on *S. aureus* CMPUJ 015 and *S. aureus* ATCC 29213 by (**A**) FMA30, (**B**) FME30. Data are represented as mean ± SD. ANOVA was used to show statistically significant differences compared to control (* *p* < 0.001).

Inhibition of biofilm was performed for the two fractions tested at sub-inhibitory concentrations. The results were expressed as the percentage of inhibition (Figure 2). An inhibition of biofilm formation higher than 80% was observed at 9 µg/mL and 120 µg/mL with fractions FMA30 and FME30, respectively.

### 2.4. Inhibition of Virulence Factors

An inhibition of hemolytic activity higher than 90% was observed using a concentration of MIC_20_ of fraction FMA30, and at the same concentration was evidenced inhibition of protease activity (Table 3). Based on the tests carried out for coagulase effect, with the FME30 fraction, there is a marked inhibition in the formation of clots when compared with the control; on the contrary, with the FMA30 fraction there was little or no inhibition (Table 4, Figure S2).

## 3. Discussion

Among the components of the mucus secretion of *A. fulica* are mucoproteins, peptides, uronic acids, glycosaminoglycans and allantoin; however, those directly related to antimicrobial activity appear to be peptides and proteins [9]. This is supported by studies that have identified peptides and proteins with antimicrobial activity against Gram-positive and Gram-negative bacteria, for example, the glycoprotein *Achacin* has antimicrobial activity against *Bacillus subtilis*, *S. aureus*, *Escherichia coli* and *Pseudomonas aeruginosa* [20,21], and the cysteine-rich peptide *mytimacin-AF*, isolated from the mucus secretion, has activity against *S. aureus*, *Bacillus megatherium*, *E. coli*, *Bacillus pyocyaneus*, *B. dysenteriae*, *Klebsiella pneumoniae* and *Candida albicans*, reporting for the first time, antifungal activity [12]. In addition, proteins of 50.81 kDa, 15 kDa and 11.45 kDa have been reported biological activity *against Streptococcus mutans* and *Aggregatibacter actinomycetemcomitans* [22].

Although there are studies that demonstrate the antimicrobial properties of the mucus secretion of *A. fulica*, little is known about the activity on resistant bacteria. A previous study carried out by our research group determined the inhibitory action of mucus secretion against *S. aureus* CMPUJ 015 [9], which was confirmed in this work.

From the results, it is considered the mucus secretion and its fractions have a bacteriostatic effect instead of bactericidal effect on *S. aureus* strains, because an increase of the culture absorbance was observed after 24 h of exposure to the fractions, indicating growth of a viable cell. However, it is necessary to carry out complementary tests to determine the mechanism of action of the components of the fractions that exert antimicrobial activity.

The ability of bacteria to form biofilms is one of the main virulence factors that interferes with the activity of antibiotics and mechanisms of immune defense response. For this reason, the inhibition of biofilm formation has become very important in the search for new strategies to combat the antimicrobial resistance issues [23]. We have not found any study evaluating the effects of mucus secretion of *A. fulica* on bacterial biofilm formation. In this study, we found that fractions isolated from mucus secretion had an inhibitory effect on the biofilm formation of both *S. aureus* strains at concentrations below the MIC_50_. This result is very important since biofilm-forming *S. aureus* strains is the major cause of infection in medical implants and wounds [2].

Since the treatment with anti-virulence agents can be an alternative to antibiotics therapy, anti-virulence effects of the fractions of *A. fulica* were also assessed. In this context, *S. aureus* produces an arsenal of virulence factors, including α-hemolysin, coagulase, and proteases, which are involved in different pathogenicity processes and biofilm formation. An inhibition of hemolytic and protease activities, using a concentration of MIC_20_ for both fractions, were determined (Table 2). Based on the tests carried out for coagulase effect, the FME30 fraction induced a strong inhibition on the formation of clots when compared to the control, whereas the FMA30 fraction showed a weak inhibitory effect.

To our knowledge, this is the first report of antibiofilm and anti-virulence effect of the mucus from *A. fulica*. Although in recent years, different studies have showed evidence of inhibition of virulence factors and biofilm formation of *S. aureus* strains, those were carried out with different compounds and natural products such as essential oils, antimicrobial peptides and nanoparticles [24,25,26,27,28,29,30,31,32], but not with gastropod mucus secretions.

Protein fractions, derived from the mucus secretion of *A. fulica,* are a rich source of antimicrobial, antibiofilm and anti-virulence bioactive molecules against *S. aureus* strains. The analysis with SDS-PAGE revealed the presence of proteins ranged from 20 to 80 kDa and by mass spectrometry analysis was identified as hemocyanin-derived peptides present in FMA30.

Most of the known AMPs come from the processing of larger inactive proteins; however, some studies suggest that biologically active proteins, such as hemocyanin [18] and hemoglobin [33], can be sources of AMPs. Hemocyanin-derived peptides with antimicrobial properties have previously been reported in shrimp [34,35,36], crayfish [18] and spiders [16]. The bibliographic review indicates that until 2015, the first AMPs derived from hemocyanin from mollusks were registered [37].

Dolashka et al., 2016, reported the antimicrobial activity of hemocyanin subunits between 45 and 65 kDa, finding inhibitory activity against Gram-positive and Gram-negative bacteria. In our work, we report the antimicrobial, antibiofilm and anti-virulence activity of semi-purified fractions containing peptides derived from hemocyanin of the gastropod *A. fulica* using subMIC, which demonstrates that the effect of the fractions is involved with the metabolism involved in biofilm formation and the different virulence factors, and is not simply due to a decrease in bacterial population.

Dolashki et al., 2020, reported three potential AMPs from the mucus secretion of the land snail *Cornu aspersum*, whose alignment in BLAST demonstrates high homology with hemocyanins isolated from snails *Helix aspersa*, *Helix pomatia* and *Helix lucorum* [38]. The authors of this study report that a comparison of the alignment of the amino acid sequence of the peptides of the mucosal secretion of *H. aspersa* with the CAMP (Collection of Anti-Microbial Peptides) databases revealed a high identity (greater than 70%) with known AMPs [38].

Probably proteolytic processes may have led to the appearance of these peptides in the mucus secretion. Some of the identified peptides contain high levels of glycine, leucine and proline residues, which are probably important for the stability of their antimicrobial activity.

The hemocyanin-derived peptides found in this study can be used for the in silico design of analogous peptides with antimicrobial activity [39]. Low molecular weight molecules, such as peptides, offer advantages over high molecular weight subunits, including greater ease in synthesis, ease of making modifications that improve their activity and ease of encapsulation or fixation to improve their bioavailability, among others [40].

## 4. Materials and Methods

### 4.1. Mucus Collection and Sample Fractionation

The specimens of *A. fulica* were collected in Floridablanca, Santander, Colombia. Snails were identified as *A. fulica* by morphological characteristics. Specimens with size of the shell between 5–12 cm were collected. The mucus secretion was obtained by direct stimulation on the foot of the snail with an electric current of 9 V at intervals of 30 to 60 s. A pool of mucus was collected in sterile Falcon tubes (50 mL).

The sample was homogenized by mixing equal volume of mucus secretion and phosphate-buffered saline (PBS) containing protease inhibitors (ethylenediaminetetraacetic acid (EDTA) 2 mM, phenylmethylsulfonyl fluoride (PMSF) 1 mM, and sodium orthovanadate 1 mM in constant agitation (150 rpm) for 24 h. The samples were centrifuged at 8000× *g* for 15 min at 4 °C. The supernatant was precipitated with ammonium sulfate at 60% to recover proteins and peptides and remove impurities. The biological extract obtained was fractionated based on their molecular size by using ultracentrifugal filters (30 kDa and 10 kDa). The fractions collected, FMA30 (fraction containing proteins and peptides with molecular size more than 30 kDa) and FME30 (fraction containing proteins and peptides with molecular size less than 30 kDa), were freeze-dried and stored at −80 °C before use.

### 4.2. SDS-PAGE Electrophoresis

In order to identify the molecular weight of the fractions obtained, SDS-PAGE electrophoresis was carried out in 14% polyacrylamide gel. In brief, the samples were mixed with Laemmli buffer (3.55 mL of type I water; 1.25 mL of 0.5 M Tris-HCl at pH 6.8; 2.5 mL of 0.5 M Tris-HCl at pH 6.8; 2.5 mL of 0.5 M Tris-HCl at pH 6.8; 2.5 mL glycerol; 2 mL 10% SDS and 0.2 mg 0.5% bromophenol blue) in 1:1 proportion and heated to 95 °C for 5 min. The first well was loaded with 5 µL of Pierce™ Unstained Protein MW Marker (Thermo Fisher Scientific, Waltham, MA, USA) and 15 µg of total protein for the sample wells. Gels were run at an initial voltage of 50 V for 20 min, then, the voltage was raised to 150 V for 85 min. The gel was removed and subjected to Coomassie R-250 staining for 1 h, and washed with decolorizing solution (50% methanol, 10% acetic acid) until the respective visualization of the bands was achieved. The gels were scanned with a ChemiDoc™ Imaging System (Bio-Rad Laboratories, Hercules, CA, USA) and analyzed with Image Lab 6.1 software (Bio-Rad Laboratories, Hercules, CA, USA).

### 4.3. Molecular Mass Analysis

To the mass spectrometry protein identification, the SDS-PAGE protein bands were excised from gels and subjected to reduction (10 mM dithiothreitol), alkylation (50 mM iodoacetamide), with subsequent overnight in-gel digestion with sequencing grade bovine trypsin (in 25 mM ammonium bicarbonate) using an automated workstation (Intavis, Cologne, Germany). The resulting peptides were submitted to nESI-MS/MS on a Q Exactive Plus^®^ mass spectrometer (Thermo Fisher Scientific, Waltham, MA, USA). A total of 10 µL of each tryptic digest were loaded on a C18 trap column (75 μm × 2 cm, 3 μm particle; PepMap, Thermo), washed with 0.1% formic acid (solution A), and separated at 200 nL/min with Easy-spray^®^ analytical column using a nano-Easy^®^ 1200 chromatograph (3 µm particle, 15 cm × 75 µm C18, Thermo Fisher Scientific, Waltham, MA, USA). A gradient from 0.1% formic acid (solution A) to 80% acetonitrile with 0.1% formic acid (solution B) was developed: 1–5% B in 1 min, 5–26% B in 25 min, 26–79% B in 4 min, 79–99% B in 1 min and 99% B in 4 min, for a total time of 35 min. MS spectra were acquired in positive mode at 1.9 kV, with a capillary temperature of 200 °C, using 1 scan at 400–1600 m/z, maximum injection time of 100 msec, AGC target of 3 × 106 and orbitrap resolution of 70,000. The top 10 ions with 2–5 positive charges were fragmented with AGC target of 1 × 105, maximum injection time of 110 msec, resolution 17,500, loop count 10, isolation window of 1.4 m/z and a dynamic exclusion time of 5 s. MS/MS spectra were processed for peptide matching with protein sequences contained in the UniProt/SwissProt database for mollusks, using PEAKS X^®^ (Bioinformatics Solutions, Waterloo, ON, Canada). Cysteine carbamidomethylation was set as a fixed modification, while deamidation of asparagine or glutamine and methionine oxidation were set as variable modifications, allowing up to 3 missed cleavages by trypsin. Parameters for match acceptance were set to FDR < 1%, and −10lgP protein score ≥ 30.

### 4.4. Bacterial Strains and Growth Conditions

*Staphylococcus aureus* ATCC 29213 strain was purchased from the American Type Culture Collection (ATCC; Rockville, MD, USA) and *Staphylococcus aureus* CMPUJ 015 strain was purchased from the Coleccion Microorganisms Pontificia Universidad Javeriana, which is a certified institution belonging to the World Federation of Culture Collection; antibiotic resistance pattern data were presented in the supplementary information (Table S1). Before being used for the antimicrobial, antibiofilm and anti-virulence assays, both strains were grown in Müller Hilton broth (MH) at 37 °C.

### 4.5. Determination of Minimum Inhibitory Concentration (MIC_50_)

The antimicrobial effects were evaluated by using the broth microdilution method described by CLSI-M07-A10-2015 [41] adapted for new antimicrobial compounds Cruz et al., 2014 [42]. The evaluation of the minimum inhibitory concentration at 50% of the microbial population (MIC_50_) was determined as follows: a culture of each microorganism was grown in MH for 12 h at 37 °C with constant agitation at 200 rpm; these were adjusted until a concentration of 1.5 × 10^8^ CFU/mL. Then, 100 µL of the inoculum was mixed with 100 µL of mucus fraction in microplates for final concentrations of 10, 20, 50, 120, 250, 500 and 1250 µg/mL. Microplates were incubated at 37 °C with constant agitation at 200 rpm. Microbial growth was measured using a Multiskan sky spectrophotometer (Thermo Labsystems Inc., Beverly, MA, USA) at 595 nm every hour for 8 h.

### 4.6. Antibiofilm Activity Assay

The evaluation of the in vitro inhibition of biofilm formation was carried out according to the method described by Molhoek et al., 2011 [43], with some modifications. Bacterial strains were grown overnight in Tryptic Soy Broth (TSB) at 37 °C and diluted in fresh medium (1:10). Then, 100 µL of cell suspension was added to sterile 96-well flat-bottom polystyrene microplates containing sub-inhibitory concentrations (subMIC) of 3, 6, 9, 12, 15 µg/mL for FMA30 and 40, 80, 120, 160, 200 µg/mL for FME30. Microplates were incubated at 37 °C for 24 h without shaking. Biofilm biomass was quantified using the crystal violet staining method. The microplates were washed three times with sterile 1 mM PBS pH 7 to remove free-floating planktonic bacteria. Then, 200 µL of 0.4% (*w/v*) crystal violet was added to each of the wells for 15 min. Crystal violet excess was eliminated by three consecutive washes with sterile 1 mM PBS pH 7; 200 µL of 30% (*v/v*) acetic acid was added to remove the adhered dye. The content of each well was transferred to a new microplate to quantify the absorbance at 595 nm using a Multiskan sky spectrophotometer (Thermo Labsystems Inc., Beverly, MA, USA).

### 4.7. Anti-Virulence Activity Assay

The possible inhibitory effect of mucus fractions on virulence factors such as hemolysin, coagulase and protease were evaluated following the method reported by Lee et al., 2014 [24], with some modifications. The microorganisms were incubated in TSB for 12 h at 37 °C with constant agitation at 200 rpm, the concentration was adjusted to 1.5 × 10^8^ CFU/mL and diluted 1:100 in TSB. This suspension and MIC_20_ of the two fractions were added to the same proportion and incubated at 37 °C with constant agitation for 12 h at 200 rpm. Finally, the supernatant was centrifuged and conserved.

Evaluation of antihemolytic effect of the mucus fractions was performed as follows: 100 µL of the supernatant and 100 µL of a suspension of human red blood cells (the blood samples were donated by the Hemocentro de Santander, samples were screened for infectious agents including HIV and hepatitis B and the donor signed informed consent) were added into a microplate, incubated for 1 h at 37 °C under agitation at 200 rpm, and centrifuged at 4000 rpm for 4 min. Supernatant was transferred to another microplate for reading at 430 nm to determine the percentage of inhibition of hemolytic activity. Assays were performed in triplicate.

The inhibitory effect on proteases was evaluated by adding 100 µL of supernatant and 100 µL of skim milk (1.25%) to a microplate, then, incubated for 1 h at 37 °C under agitation at 200 rpm, and centrifuged at 4000 rpm for 4 min. The supernatant was transferred to another microplate for reading at 600 nm to determine the percentage of inhibition of protease activity. Assays were performed in triplicate.

The evaluation of the inhibitory effect on coagulase enzyme was performed by taking 100 µL of supernatant and 200 µL of citrated plasma, incubated for 12 h at 37 °C, and then visualized for the total presence, partial presence or absence of the colt. Assays were performed in triplicate.

### 4.8. Data Analysis

Unless otherwise stated, statistical tests were carried out with SigmaPlot 12.0 (SYSTAT Software Inc., San Jose, CA, USA). All the experiments were performed by triplicates and one-way analysis of variance (ANOVA) was used to analyze the differences among the treatments. In all cases, the level of significance was 0.05. Assumption of normality and equally of variances of data was previously tested using Shapiro–Wilk and Levene’s test, respectively. 

## 5. Conclusions

*Staphylococcus aureus* is classified by WHO as one of the most important microorganisms for the development of new pharmacological alternatives to control it. This study demonstrates for the first time that the fractions of the mucus secretion of *Achatina fulica* exhibited anti-virulence activity against *S. aureus* strains at sub-inhibitory concentrations.

In the present study, both fractions demonstrated antibacterial, antibiofilm and anti-virulence activity against *Staphylococcus aureus* CMPUJ 015 and *Staphylococcus aureus* ATCC 29213. The fraction FMA30 demonstrated the highest antibacterial and antibiofilm activity against *S. aureus* ATCC 29213 and *S. aureus* CMPUJ 015, this is mostly due to the purification and precipitation process with ammonium sulfate, which allowed eliminating impurities and concentrating the proteins and peptides present in the secretion, demonstrating that the antimicrobial activity of mucus is related to the protein material present as mentioned by different authors. Additionally, both fractions showed a highest antihemolytic effect against the *S. aureus* strains and a highest antiprotease effect against *S. aureus* strains; in contrast, only FME30 had a high anticoagulase effect.

Further studies are needed to elucidate the mechanisms of action of the *A. fulica* fractions and to determine the compounds related to the biological activity evaluated. Metabolomic analyses are underway to determine the metabolic pathways affected in the bacteria when treated with these fractions, as well as the characterization of the peptides and proteins present in the secretion.

## Figures and Tables

**Table 1:** Peptides that are part of hemocyanin identified in fraction FMA30 of the mucus secretion of *A. fulica.*

#	Sequence	Origin	ID
1	IYSRPADTFDYRN	Hemocyanin alphaD OS = *Cornu aspersum* OX = 6535 PE = 2 SV = 1	A0A3G2VFQ5
2	RLLTVQAENALRKH	Hemocyanin alphaD OS = *Cornu aspersum* OX = 6535 PE = 2 SV = 1	A0A3G2VFQ5
3	RKPLQPFQDKT	Hemocyanin alphaD OS = *Cornu aspersum* OX = 6535 PE = 2 SV = 1	A0A3G2VFQ5
4	RLHGIGVSADVRV	Hemocyanin alphaD OS = *Cornu aspersum* OX = 6535 PE = 2 SV = 1	A0A3G2VFQ5
5	REMPWAYERL	Hemocyanin alphaD OS = *Cornu aspersum* OX = 6535 PE = 2 SV = 1	A0A3G2VFQ5
6	RKPLQPFQDKK	Hemocyanin alphaD OS = *Helix pomatia* OX = 6536 PE = 2 SV = 1	A0A3G2VHR9
7	RYSRPADTFDYRN	Hemocyanin 1 OS = *Lymnaea stagnalis* OX = 6523 PE = 2 SV = 1	A0A3G2VM51
8	RAIDAFDYDRL	Hemocyanin 1 OS = *Lymnaea stagnalis* OX = 6523 PE = 2 SV = 1	A0A3G2VM51
9	KYDVTNVFNKL	Hemocyanin 1 OS = *Lymnaea stagnalis* OX = 6523 PE = 2 SV = 1	A0A3G2VM51
10	KEMPWAYERI	Hemocyanin 1 OS = *Lymnaea stagnalis* OX = 6523 PE = 2 SV = 1	A0A3G2VM51
11	SGRVEFEHVDTERD	Hemocyanin alphaN-subunit (Fragment) OS = *Helix lucorum* OX = 31,229 PE = 2 SV = 1	G3FPE7
12	RYDVTNVFNKL	Hemocyanin alphaN-subunit (Fragment) OS = *Helix lucorum* OX = 31,229 PE = 2 SV = 1	G3FPE7
13	RLYVVQLEQALKEKG	Hemocyanin 1 OS = *Lymnaea stagnalis* OX = 6523 PE = 2 SV = 1	A0A3G2VM51
14	DPLFLLHHSNVDRQ	Hemocyanin 1 OS = *Lymnaea stagnalis* OX = 6523 PE = 2 SV = 1	A0A3G2VM51
15	KYSRPIDTFDYRN	Hemocyanin alphaD OS = *Cornu aspersum* OX = 6535 PE = 2 SV = 1	A0A3G2VHN3
16	RLLTVQAENALRN	Hemocyanin alphaD OS = *Cornu aspersum* OX = 6535 PE = 2 SV = 1	A0A3G2VHN3
17	RIYIVVEDH	Hemocyanin alphaD OS = *Cornu aspersum* OX = 6535 PE = 2 SV = 1	A0A3G2VHN3
18	RAIDAFDYDRF	Hemocyanin 1 OS = *Aplysia californica* OX = 6500 PE = 2 SV = 1	A0A3G9M8B7
19	RLLTVQAENALRR	Hemocyanin 1 OS = *Aplysia californica* OX = 6500 PE = 2 SV = 1	A0A3G9M8B7
20	KVAGEDAVTTRD	Hemocyanin alphaD OS = *Cornu aspersum* OX = 6535 PE = 2 SV = 1	A0A3G2VFQ5

**Table 2:** Minimum inhibitory concentration 50 (MIC_50,_ µg/mL) and sub-inhibitory concentrations (MIC_20,_ µg/mL) of the two fractions on *S. aureus* CMPUJ 015 and *S. aureus* ATCC 29213.

Mucus Fraction	Antimicrobial Activity			
	*S. aureus* CMPUJ 015		*S. aureus* ATCC 29213	
	MIC_20_	MIC_50_	MIC_20_	MIC_50_
FMA30	10	25	12	125
FME30	120	500	250	750

**Table 3:** Inhibition of hemolysin and protease production by *S. aureus* strains.

Mucus Fraction	*S. aureus* CMPUJ 015		*S. aureus* ATCC 29213	
	Hemolytic Activity Inhibition (%)	Protease Activity Inhibition (%)	Hemolytic Activity Inhibition (%)	Protease Activity Inhibition (%)
FMA30	98.60 ± 1.44	73.55 ± 2.08	97.43 ± 2.97	<20
FME30	96.69 ± 1.89	80.34 ± 3.72	75.74 ± 3.39	31.46 ± 6.15

**Table 4:** Inhibition of the coagulase production of *S. aureus* strains.

Mucus Fraction	*S. aureus* CMPUJ 015	*S. aureus* ATCC 29213
FMA30	+++	++
FME30	+	+

(+++) Total, (++) partial, (+) minimum presence of clots.

## Data Availability

Data are contained within the article.

## Supplementary Materials

The following are available online at https://www.mdpi.com/article/10.3390/antibiotics10121548/s1, Figure S1: Growth kinetics assay of antimicrobial effect of FME10 against (A) *S. aureus* ATCC 29213 and (B) *S. aureus* CMPUJ 015; Figure S2: Inhibition of the coagulase production of *S. aureus* strains. (a) *S. aureus* CMPUJ 015 (b) *S. aureus* ATCC 29213; Table S1 antibiogram of *S. aureus* strains.
