# Supplementary material for: Antibacterial, Antibiofilm and Anti-Virulence Activity of Biactive Fractions from Mucus Secretion of Giant African Snail Achatina fulica against Staphylococcus aureus Strains
Source: Antibiotics (Basel). 2021 Dec 17;10(12):1548. doi: 10.3390/antibiotics10121548 (PMC8698528; doi:10.3390/antibiotics10121548)
Supplement: Supplementary file 1 [file antibiotics-10-01548-s001.zip › antibiotics-1494770sup-final.pdf]

Supporting information

Antibacterial, Antibiofilm and Anti-virulence Activity of Biactive Fractions from Mucus Secretion of Giant African Snail *Achatina fulica* against *Staphylococcus aureus* Strains

Libardo Suárez [1,2], Andrés Pereira [2], William Hidalgo [1,*] and Nelson Uribe [2]

[1] Grupo de Investigación en Bioquímica y Microbiología (GIBIM), Escuela de Química, Universidad Industrial de Santander, Edificio Camilo Torres 202, Bucaramanga 680002, Colombia; libardo2178128@correo.uis.edu.co
[2] Grupo de Inmunología y Epidemiología Molecular (GIEM), Escuela de Microbiología, Facultad de Salud, Universidad Industrial de Santander, Bucaramanga 680002, Colombia; andres.pereira@correo.uis.edu.co (A.P.); nelurdel@uis.edu.co (N.U.)
* Correspondence: whidalgo@uis.edu.co; Tel.: +57-(7)-634-40-00 (ext. 2025)

A.

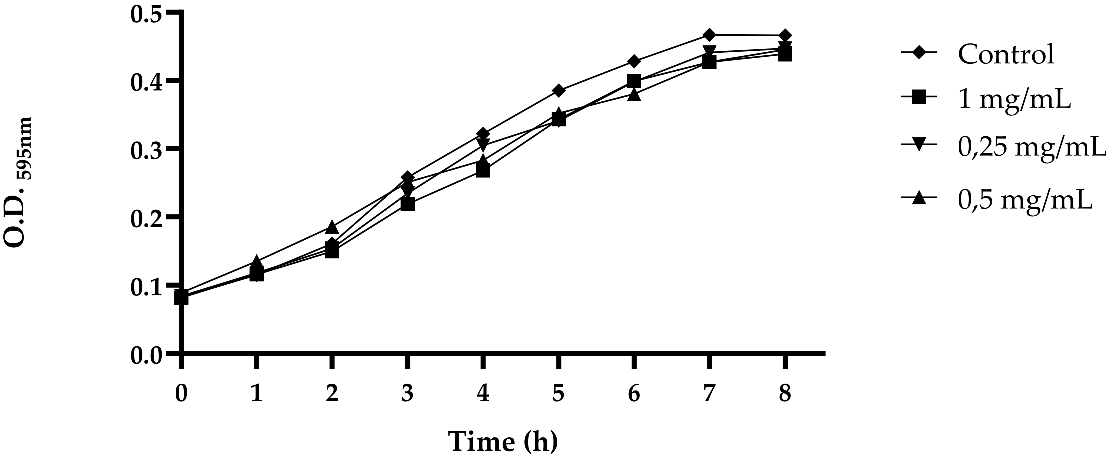

B.

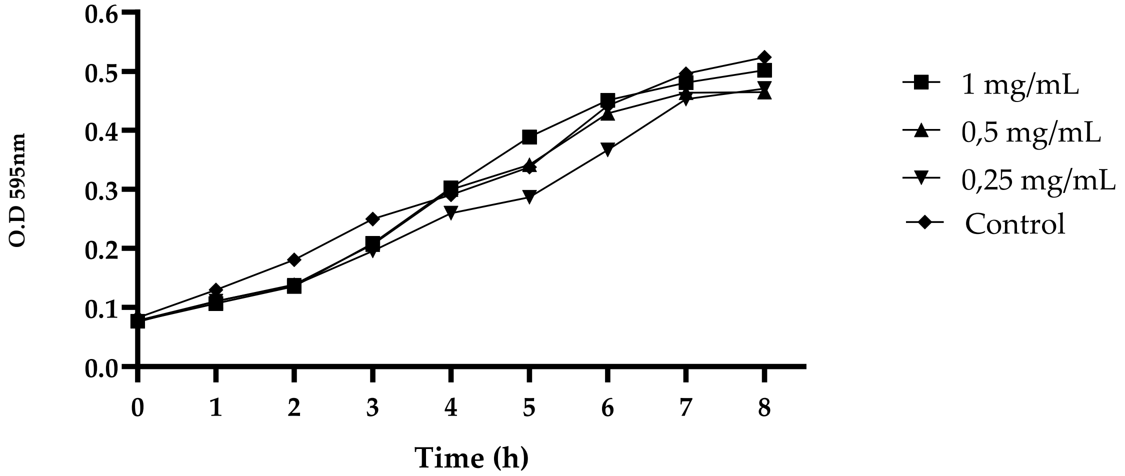

Figure S1. Growth kinetics assay of the antimicrobial effect of FME10 against (**A**) *S. aureus* ATCC 29213 and (**B**) *S. aureus* CMPUJ 015.

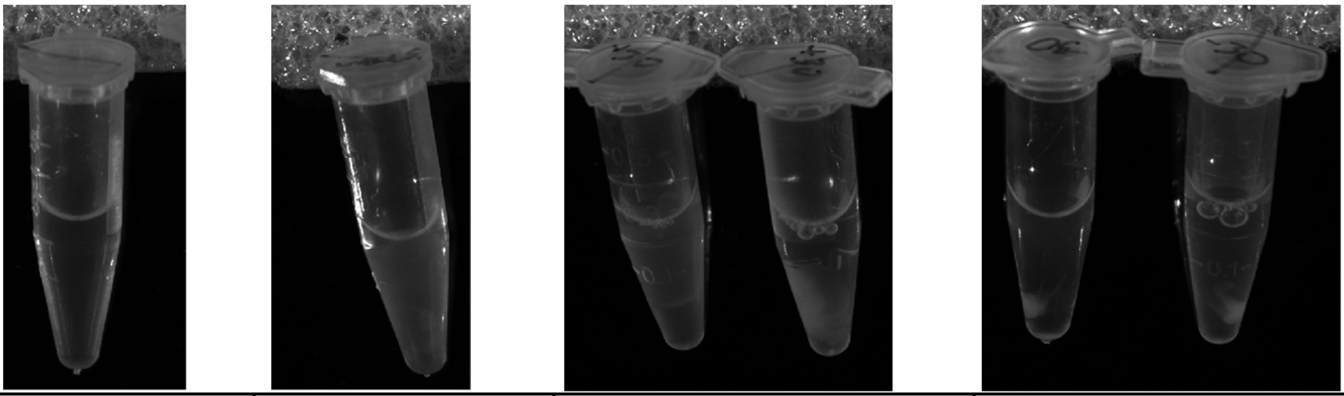

Negative Control (TSB culture media)	Positive control (*S. aureus* CMPUJ 015 growth in TSB culture media)	FMA30 (*S. aureus* CMPUJ 015 treated with FMA30)	FME30 (*S. aureus* CMPUJ 015 treated with FME30)
-	+++	+++	+

(**a**)

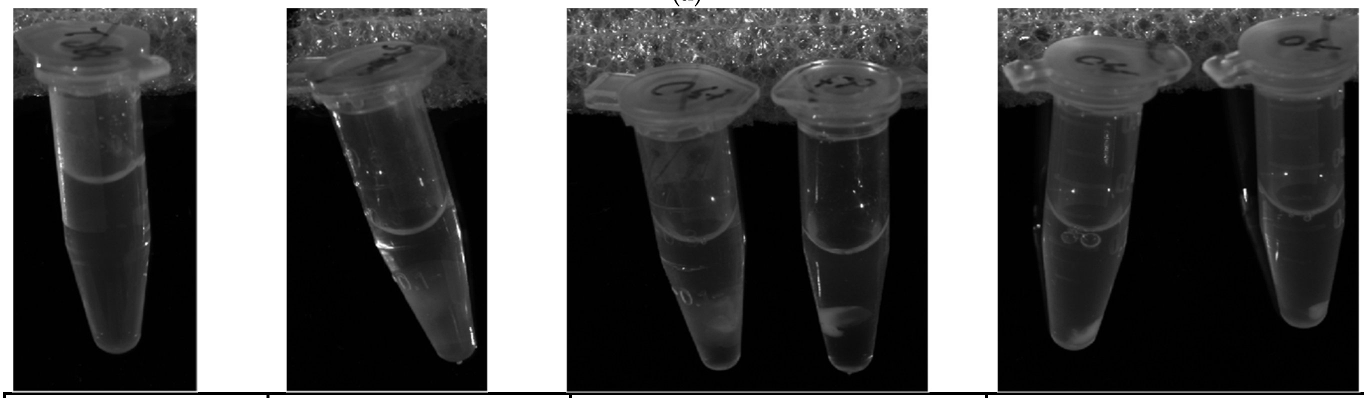

Negative Control (TSB culture media)	Positive control (*S. aureus* ATCC 29213 growth in TBS culture media)	FMA30 (*S. aureus* ATCC 29213 treated with FMA30)	FME30 (*S. aureus* ATCC 29213 treated with FME30)
-	+++	++	+

(**b**)

Figure S2. Inhibition of the coagulase production of *S. aureus* strains. (**a**) *S. aureus* CMPUJ 015 (**b**) *S. aureus* ATCC 29213. TBS (Tryptic Soy Broth) culture media. (+++) Total, (++) partial, (+) minimum presence of clots.

Table S1. Antibiogram of *S. aureus* strains. The clinical tests were carried out by the service of Merced Lab, Bucaramanga, Colombia.

Antibiotic	*S. aureus* CMPUJ 015		*S. aureus* ATTC 29213	
	MIC (ug/mL)	Interpretation	MIC (ug/mL)	Interpretation
Amox/A Clav	<=4/2	S	<=4/2	S
Amp/Sulbactam	>16/8	R	16/8	I
Ampicillin	>8	R	>8	R
Cefazolin	<=4	S	<=4	S
Ciprofloxacin	<=1	S	<=1	S
Clindamycin	1	I	1	I
Daptomycin	1	S	1	S
Erythromycin	>4	R	2	I
Gentamicin	<=4	S	<=4	S
Induction Clindamycin	<=4/0.5	NEG	<=4/0.5	NEG
Levofloxacin	<=1	S	<=1	S
Linezolid	4	S	4	S
Moxifloxacin	<=0.5	S	<=0.5	S
Nitrofurantoin	<=32		<=32	
Oxacillin	2	S	1	S
Penicillin	>8	R	>8	R
Rifampicin	<=1	S	<=1	S
Cefoxitin Screening	<=4	NEG	<=4	NEG
Synercid	<=1	S	<=1	S
Tetracycline	>8	R	>8	R
Trimet/Sulfa	<=0.5/9.5	S	<=0.5/9.5	S
Vancomycin	2	S	2	S

S: sensible; I: intermediate sensitivity; R: resistant; NEG: Negative.
